# A screening method for aortic valve disease based on spectral analysis of a single-channel electrocardiogram using machine learning algorithms

**DOI:** 10.3389/fcvm.2026.1969401

**Published:** 2026-10-08

**Authors:** Natalia Kuznetsova, Daniil Zelenov, Aleksandra Bogdanova, Ines Dhif, Ekaterina Shobonova, Anna Alekseeva, Dinara Mesitskaya, Abram Syrkin, Roman Komarov, Philippe Kopylov, Petr Chomakhidze

**Affiliations:** 1 Institute of Personalized Cardiology of Biomedical Science and Technology Park, Sechenov First Moscow State Medical University (Sechenov University), Moscow, Russia; 2 Department of Cardiology, Functional and Ultrasound Diagnostics of N.V. Sklifosovsky Institute for Clinical Medicine, Sechenov First Moscow State Medical University (Sechenov University), Moscow, Russia; 3 Russian Medical Academy of Continuous Professional Education, Moscow, Russia; 4 Department of Cardiovascular Surgery and Invasive Cardiology, Sechenov First Moscow State Medical University (Sechenov University), Moscow, Russia

**Keywords:** aortic regurgitation, aortic stenosis, artificial intelligence, ECG, machine learning, spectral analysis

## Abstract

**Background:**

Given the high prevalence of aortic heart disease and the importance of early diagnosis, there is a need to develop a simple screening method to identify significant aortic heart disease based on available biosignals, one of which is a single-channel electrocardiogram (ECG).

**Aim:**

We aimed to develop a machine learning algorithm for analyzing single-channel electrocardiograms to identify patients with moderate to severe aortic stenosis and regurgitation, as well as significant aortic valve disease.

**Methods:**

Patients (*n* = 2,503) underwent echocardiographic examination and single-channel I-lead ECG recording using a portable electrocardiograph. Moderate to severe aortic stenosis on echocardiography was present in 62 (2.4%) patients, and moderate to severe aortic regurgitation was present in 157 (6.3%). Single-channel ECG analysis was performed using advanced signal processing and machine learning techniques.

**Results:**

Both models were evaluated under an identical 10-fold group cross-validation framework. For detecting moderate or severe aortic stenosis, diagnostic accuracy was 0.650 (Se 57.9%, Sp 65.0%) for Balanced Random Forest and 0.662 (Se 46.4%, Sp 66.5%) for XGBoost. For moderate or severe aortic regurgitation, accuracy was 0.726 (Se 60.5%, Sp 73.3%) for Balanced Random Forest and 0.712 (Se 61.7%, Sp 71.9%) for XGBoost. For significant aortic valve disease, accuracy was 0.724 (Se 61.1%, Sp 73.4%) for Balanced Random Forest and 0.691 (Se 67.8%, Sp 69.2%) for XGBoost.

**Conclusions:**

The results indicate good diagnostic accuracy and potential applicability for screening for significant aortic heart disease using single-channel ECG parameters analyzed with modern signal processing and machine learning techniques.

**Clinical Trial Registration**: https://clinicaltrial.gov/, identifier NCT07099417.

## Graphical Abstract

**Figure:**
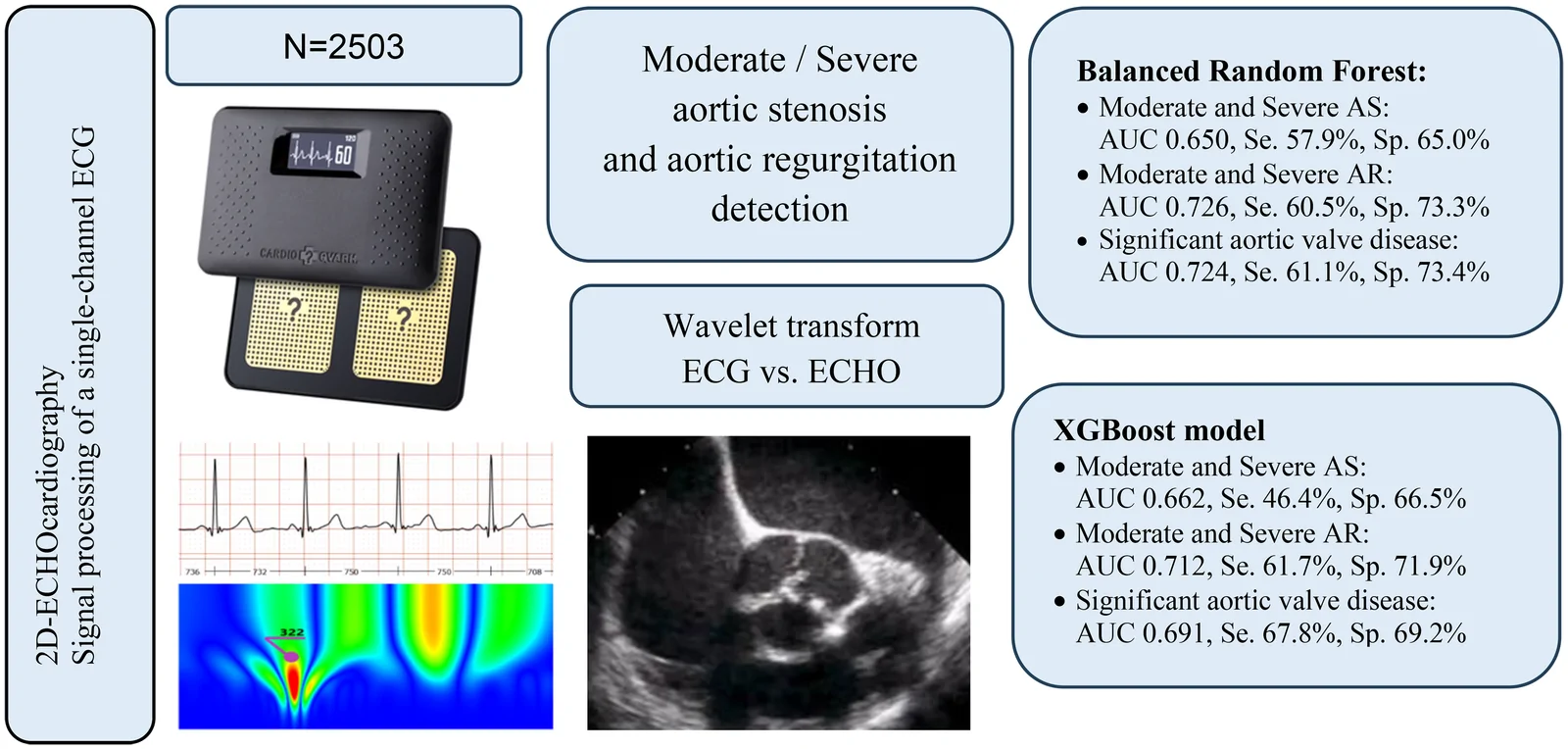

## Introduction

The prevalence of valvular heart disease (VHD) in developed countries is approximately 2.5% (1, 2).

Among VHDs, the most common are aortic stenosis (AS) and mitral regurgitation (MR), followed by aortic regurgitation (AR), mitral stenosis (MS), mitral valve prolapse, and primary tricuspid regurgitation (3). In this article, we discuss aortic valve disease (aortic stenosis and aortic regurgitation) as among the most common and severe forms of valvular heart disease.

According to data published in 2010, the prevalence of AS among patients aged 65 years and older was approximately 40 million people (2% of people over 65% and 12% of those over 75) (4, 5). It is expected that by 2030, this number will increase to 72 million people (5, 6). According to data published in 2006, clinically significant aortic valve disease affected 4.2 to 5.6 million adults in the United States (7).

The estimated prevalence of AR in the Framingham study is 4.9%, with moderate or severe AR occurring in 0.5% of the study population (8). The prevalence and severity of AR, as with other valvular pathologies, increase with age, peaking at 40–60 years of age. The annual mortality rate in symptomatic patients with severe AR who develop left ventricular (LV) dysfunction was reported to be 25% (8). These data highlight the need for timely diagnosis of heart defects to facilitate treatment and reduce the risk of complications.

Heart diseases are often detected at late stages, when patients already have symptoms that prompt diagnostic testing. It should be noted that auscultation alone does not always detect heart disease (9, 10). The specificity of these symptoms is also low (11, 12). A meta-analysis published in 2022 showed that the average sensitivity for detecting AS during auscultation ranged from 72% to 97%, while the sensitivity for detecting AR was approximately 80% according to several studies, which is insufficient for effective screening (12).

Echocardiography (ECHO) is the primary diagnostic tool for detecting valvular heart disease (13). However, ECHO is a labor-intensive, time-consuming, and expensive test, making it less accessible as a large-scale screening tool. Therefore, ECHO is more often performed in patients with suspected severe heart disease rather than in asymptomatic patients.

Thus, there is a great need to develop an affordable method for large-scale screening. The most accessible and inexpensive method for screening for heart disease is electrocardiography; however, in its usual form, it does not detect valvular heart disease. Currently, a single-channel electrocardiogram (ECG) can be recorded using various portable devices, including watches, phones, and other devices.

The aim of this study was to develop an artificial intelligence-enabled single-channel electrocardiogram (AI-ECG) processing algorithm for use as a screening tool for the detection of significant aortic valve disease (moderate to severe aortic stenosis and/or aortic regurgitation).

## Methods

This protocol was conducted in accordance with the provisions of the Declaration of Helsinki and was approved at a meeting of the local ethics commission of Sechenov University (No. 29-24, dated 05 December 2024). The study was registered on the ClinicalTrials.gov website (ID NCT07099417).

The Flow diagram is shown in Figure 1.

**Figure 1:**
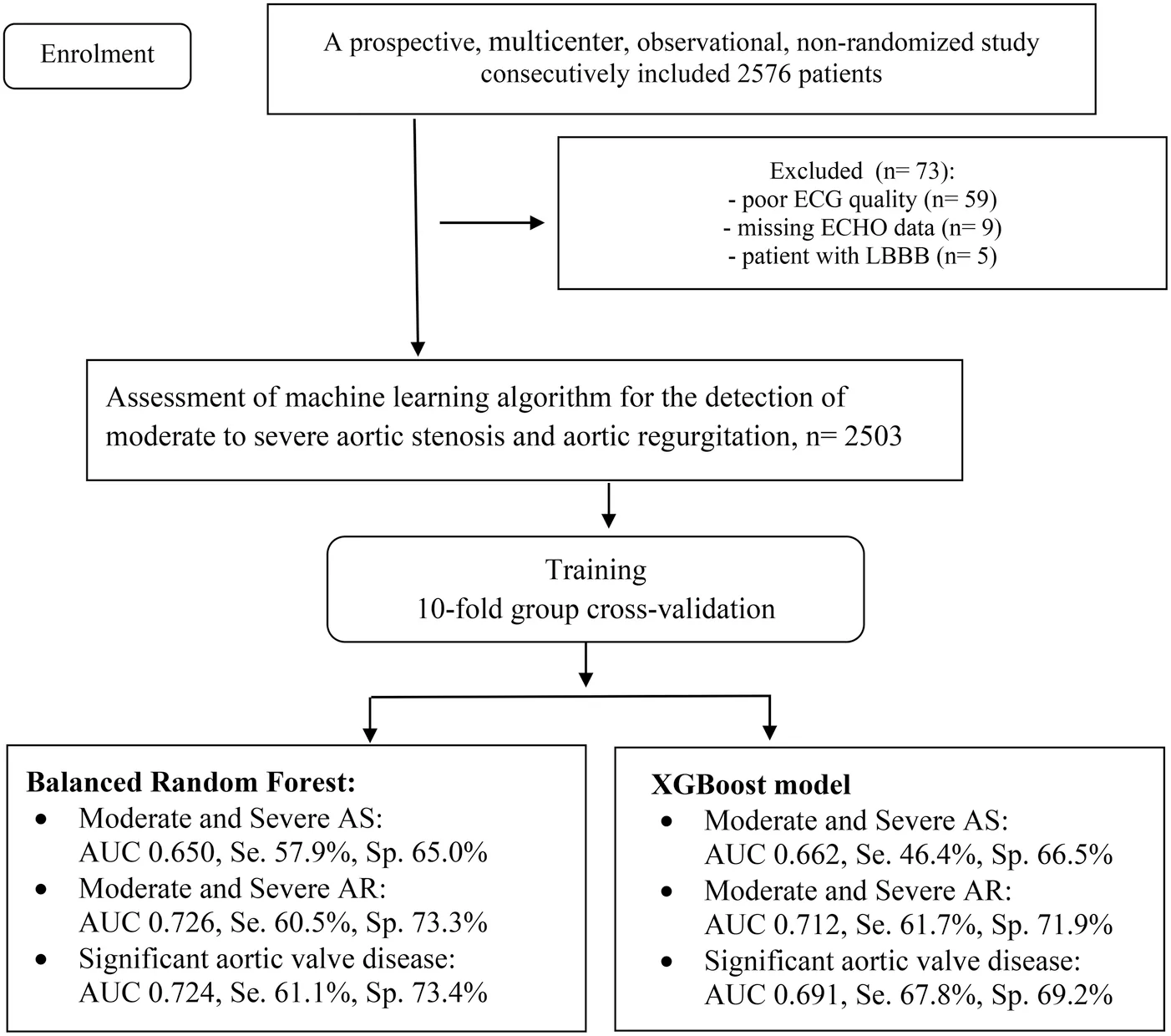
Flow diagram indicating the study plan, patient selection criteria, machine learning methods used, and the results obtained. ECG, electrocardiogram; ECHO, echocardiogram; LBBB, left bundle brunch block; AS, aortic stenosis; AR, aortic regurgitation; AUC, area under curve; Se, sensitivity; Sp, specificity.

### Cohort identification

The study included 2576 patients aged 18 years and older who were admitted as outpatients to Clinical Hospital No. 1 of Sechenov University or hospitalized in the cardiology department of the same hospital, as well as patients from City Clinical Hospital No. 1 of Moscow, between December 2024 and April 2026. This was a prospective, multicenter, observational, non-randomized study. Each participant signed an informed consent form.

Inclusion criteria: age 18 years or older; consent to participate in the study; examination, including expert echocardiography.

Non-inclusion criteria: lack of informed consent; previous heart valve surgery; known pathology that did not allow recording an ECG of satisfactory quality (WPW syndrome, pacemaker, Parkinson's disease, etc.).

Exclusion criteria: refusal to continue participation in the study; unsatisfactory ECG quality; unsatisfactory echocardiography quality; complete left bundle branch block; obvious ECG pathology that did not allow the required analysis to be performed.

A total of 73 patients were excluded due to unsatisfactory ECG quality (*n* = 59) or echocardiography quality (*n* = 9), as well as 5 patients with newly identified complete left bundle branch block.

The final cohort (*n* = 2,503) was stratified into training and testing subsets at an 80/20 ratio using GroupShuffleSplit, with groups formed based on a composite patient identifier. This approach ensured that all observations related to a single patient were included in either the training or testing set, preventing artificially inflated metrics due to correlated measurements. The frequency of significant aortic valve disease was maintained in the test sample during repeated cross-validation.

### Transthoracic echocardiogram and electrocardiogram data

Each participant underwent a brief medical history and a general examination. Each patient underwent a single transthoracic echocardiographic examination performed by two independent examiners. The examination was performed using the GE HealthCare VIVID E95 and GE VIVID-7 systems, assessing aortic stenosis and aortic regurgitation in accordance with current guidelines (14). Immediately after the ECHO, a single-channel ECG was recorded in the first standard lead at rest for 1 min using a CardioQVARK portable electrocardiograph (Moscow, Russia), registered in the Russian Federation (registration certificates issued by the Federal Service for Surveillance in Healthcare of the Russian Federation dated 15 February 2019, No. RZN 2024/8124, and 05 June 2024, No. RZN 2024/22855).

In this work we used the following ECG parameters:

- Amplitude parameters (JA, TA, ect.);
- Spectral integrals of energy of R and T waves;
- ECG time markers: Tpeak, Speak, Rpeak, ect.;
- Spectral integral (QRSE1, QRSE2);
- Indices of asymmetry
- QTc duration
- Ect.

Of the patients who underwent ECHO, we included only those for whom the following parameters were available: for aortic stenosis, mean transaortic pressure gradient or aortic valve area (AVA); and for aortic regurgitation, vena contracta (VC), the ratio of the diameter of the regurgitant flow to the diameter of the left ventricular outflow tract (LVOT), and pressure half-time (PHT) (14).

Peak velocity and mean pressure gradient were acquired by continuous-wave Doppler from all available transducer positions to obtain the highest values. AVA was calculated using the continuity equation. LVOT velocity was measured by pulsed-wave Doppler. AS severity was classified as moderate when the mean gradient was 20–39 mmHg or AVA was 1–1.2 cm^2^, and as severe when the mean gradient was ≥40 mmHg, Vmax was ≥4.0 m/s, or AVA was ≤1 cm^2^ (14). In cases of reduced ejection fraction, the severity of aortic stenosis was assessed by AVA (14).

To assess aortic regurgitation, we measured VC and the ratio of the diameter of the regurgitant flow to the diameter of the LVOT in the parasternal long-axis view. PHT was acquired by continuous-wave Doppler from all available transducer positions to obtain the highest values.

Digital ECG analysis yielded more than 200 ECG parameters that are not accessible to routine human analysis, such as the slope and angles of different segments of the cardiac cycle, geometric parameters, and others. The planned diagnostic accuracy of the algorithm was more than 75%. The physicians performing the ECHO were blinded to the single-lead ECG findings.

The primary outcome measure was the ability of ECGs analyzed using machine learning methods to detect moderate to severe aortic stenosis, moderate to severe aortic regurgitation, and the combined outcome of significant aortic valve disease (including moderate to severe aortic stenosis and regurgitation).

Features with a completeness rate below 60% were excluded to minimize the impact of missing data on model quality. The candidate feature pool comprised 54 demographic, anthropometric, and electrocardiographic variables (7 clinical/demographic and 47 ECG-derived parameters); after applying the completeness filter, all 54 features were retained for the Balanced Random Forest model.

### Statistical analysis

Statistical processing was performed using Python 3.10 with specialized libraries for data analysis and machine learning: pandas, numpy, scipy, scikit-learn, statsmodels, missingno, xgboost, and snfpy. For quantitative variables, the distribution type (using the Shapiro–Wilk test), mean, standard deviation, median, and interquartile range were determined. For categorical features, the proportions and absolute numbers of values were determined. Comparative analysis between independent groups with non-normal distributions was performed using the non-parametric Mann–Whitney *U*-test. For normally distributed features, Student's *t*-test for independent samples was used. When comparing three or more groups, one-way analysis of variance (ANOVA) or the non-parametric Kruskal–Wallis test was used. To correct for multiple comparisons, the Benjamini-Hochberg correction method was applied (multipletests, statsmodels). Correlation analysis of continuous variables was performed using Pearson's correlation coefficient (for normal distributions) and Spearman's correlation coefficient (for deviations from normality).

## Machine learning

### Class balancing

Due to a significant class imbalance (predominance of patients without significant pathology), various strategies were used depending on the model. For the Balanced Random Forest-based model, the BalancedRandomForestClassifier algorithm was used, which implements internal balancing through stratified bootstrap sampling and class weighting. This approach allows the model to pay more attention to the minority class without changing the size of the training set.

### Model 1: balanced random forest

To solve the binary classification problem, the Balanced Random Forest ensemble algorithm was used, combining the advantages of a random forest and built-in class balancing.

The generalization ability of the model was assessed using 10-fold group cross-validation (GroupKFold, n_splits = 10). For each iteration, the following quality metrics were calculated: area under the receiver operating characteristic (AUC-ROC), area under the precision-recall curve (PR-AUC), sensitivity, specificity, positive and negative predictive value (PPV, NPV), and the Youden index for selecting the optimal classification threshold.

The optimal probability threshold for binary classification was determined by maximizing the Youden index (Youden's Index = Sensitivity + Specificity − 1) over a threshold range from 0.01 to 0.99. This approach ensures a balance between the proportion of correctly identified positive and negative cases, which is consistent with the objectives of screening diagnostics.

In the Balanced Random Forest-based model, feature selection was performed using the SelectFromModel algorithm implemented in the scikit-learn library, with RandomForestClassifier (n_estimators = 1,000, random_state = 42) as the base estimator. For each cross-validation fold, no more than 10 features with the highest importance were retained (max_features = 10). Feature selection was performed strictly within the training portion of each fold to prevent information leakage. Model hyperparameters were: n_estimators = 2,000, max_depth = 12, min_samples_split = 6, min_samples_leaf = 3, sampling_strategy = 'auto', random_state = 42.

### Model 2. gradient boosting (XGBoost)

The second model was implemented using the XGBoost algorithm with parameters optimized for handling imbalance. XGBoost was trained and evaluated using the same 10-fold group cross-validation (GroupKFold, n_splits = 10) as the Balanced Random Forest model, ensuring direct comparability between the two algorithms. For both models, the classification threshold was selected by nested cross-validation (GroupKFold, 5 inner folds) performed strictly within the training portion of each outer fold, and then applied unchanged to the held-out validation fold, preventing information leakage between threshold selection and performance estimation. In addition to the Youden-optimal threshold, a second, pre-specified operating point was evaluated, fixing sensitivity at ≥90% and maximizing specificity subject to this constraint, reflecting the priority given to minimizing missed cases in a screening context.

The accuracy of the models did not vary significantly with patient age, which is important for widespread use.

## Results

### Study population

The study included 2,576 patients aged 18 years and older. A total of 73 patients were excluded due to unsatisfactory ECG quality (*n* = 59) or ECHO quality (*n* = 9), as well as 5 patients with identified complete left bundle branch block. The final cohort included 2,503 participants. The most significant clinical characteristics of all patients included in the study are presented in Table 1. The training and test groups were analyzed using cross-validation only in the absence of statistically significant differences in the presented key clinical characteristics.

**Table 1:** Clinical characteristics of all patients, included in the study, *n* = 2,503.

Characteristics	*n* (%)
Age	67,6 ± 15,4
Men	1,267 (50.6%)
Smoking for ⩾5year prior to inclusion	599 (23.9%)
Diabetes mellitus	440 (17.6%)
Hypertension	1,870 (74.7%)
Stage 1	294 (11.7%)
Stage 2	644 (25.7%)
Stage 3	932 (37.2%)
Ischemic heart disease	535 (21.4%)
Atrial fibrillation at the time of ECG registration	126 (5.0%)
AR	
Mild AR	560 (22.4%)
Moderate AR	140 (5.6%)
Severe AR	17 (0.67%)
AS	
Mild AS	123 (4.9%)
Moderate AS	46 (1.8%)
Severe AS	16 (0.6%)
MR	
Mild MR	1,480 (59.1%)
Moderate MR	520 (20.8%)
Severe MR	40 (1.6%)
LV ejection fraction <52% for men and	306 (12.2%)
LV ejection fraction <54% for women	
LV ejection fraction <40%	102 (4.1%)
Myocardial infarction	254 (10.1%)
Heart Failure	
NYHA functional class I	133 (5.3%)
NYHA functional class II	403 (16.1%)
NYHA functional class III	79 (3.2%)
NYHA functional class IV	2 (0.08%)

AS, aortic stenosis; AR, aortic regurgitation; MR, mitral regurgitation; ECG, electrocardiogram; LV, left ventricular; NYHA, New York Heart Association.

The Balanced Random Forest classifier's performance was assessed using 10-fold group cross-validation (GroupKFold, n_splits = 10). The training subset sizes ranged from 2,239 to 2,253 observations, and the validation subset sizes ranged from 248 to 251. All folds were confirmed to be free of patient identifier leakage. Values are presented as mean ± standard deviation across 10 folds.

### Balanced random forest model (10-fold group CV)

AUC-ROC values ranged from 0.675 to 0.746, indicating a moderate ability of the model to discriminate between patients with and without pathology Table 2.

**Table 2:** Quality metrics of models (10-fold group CV).

Target variable	Balanced random forest	XGBoost
Moderate and Severe AS	0.675 ± 0.130	0.634 ± 0.155
Moderate and Severe AR	0.736 ± 0.064	0.741 ± 0.068
Significant aortic valve disease	0.746 ± 0.045	0.741 ± 0.052

AS, aortic stenosis; AR, aortic regurgitation.

The highest value was obtained for the composite objective “Significant aortic valve disease” (AUC-ROC = 0.746 ± 0.045) Figures 2–4.

**Figure 2:**
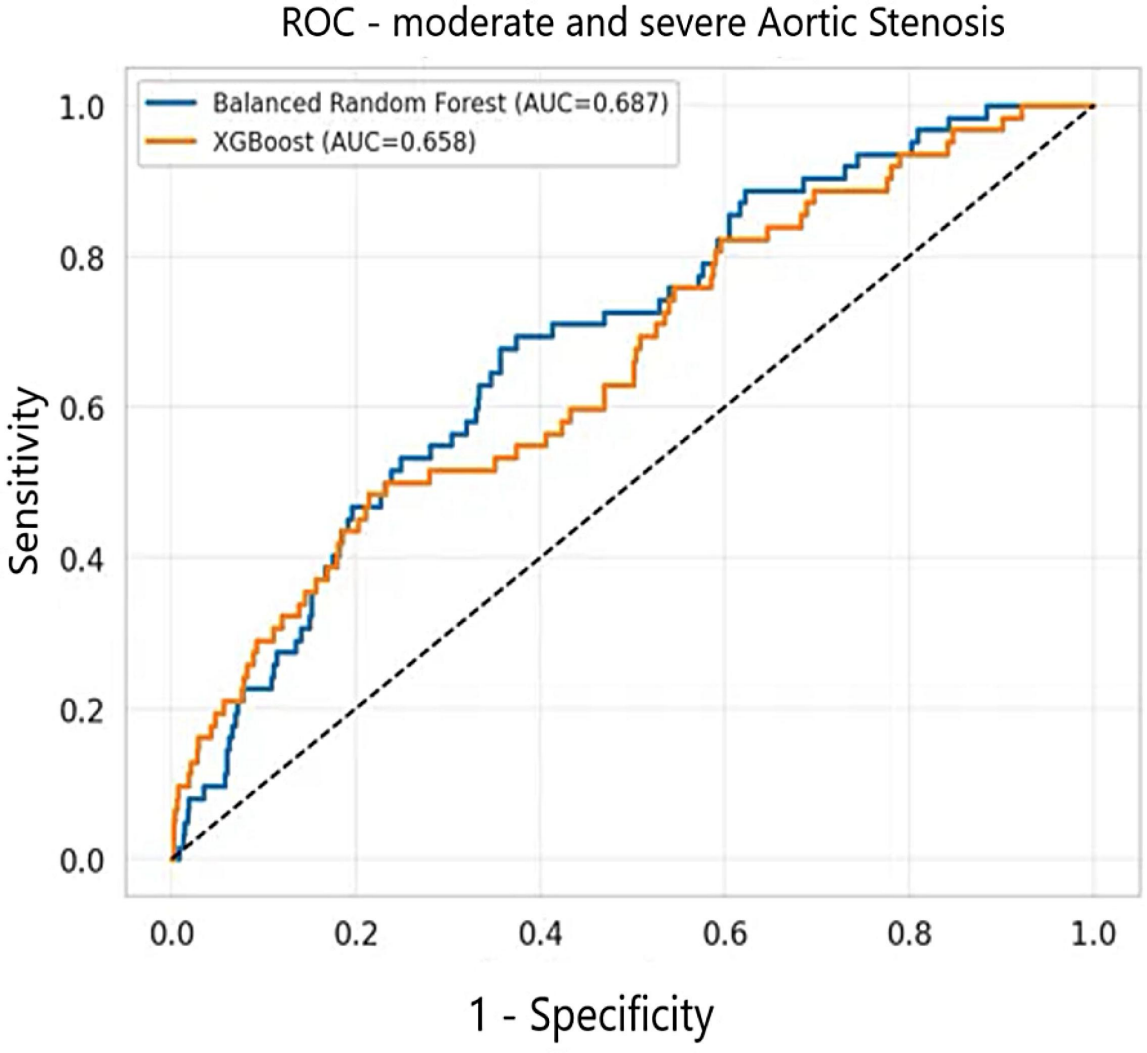
ROC curve. Moderate and severe aortic stenosis.

**Figure 3:**
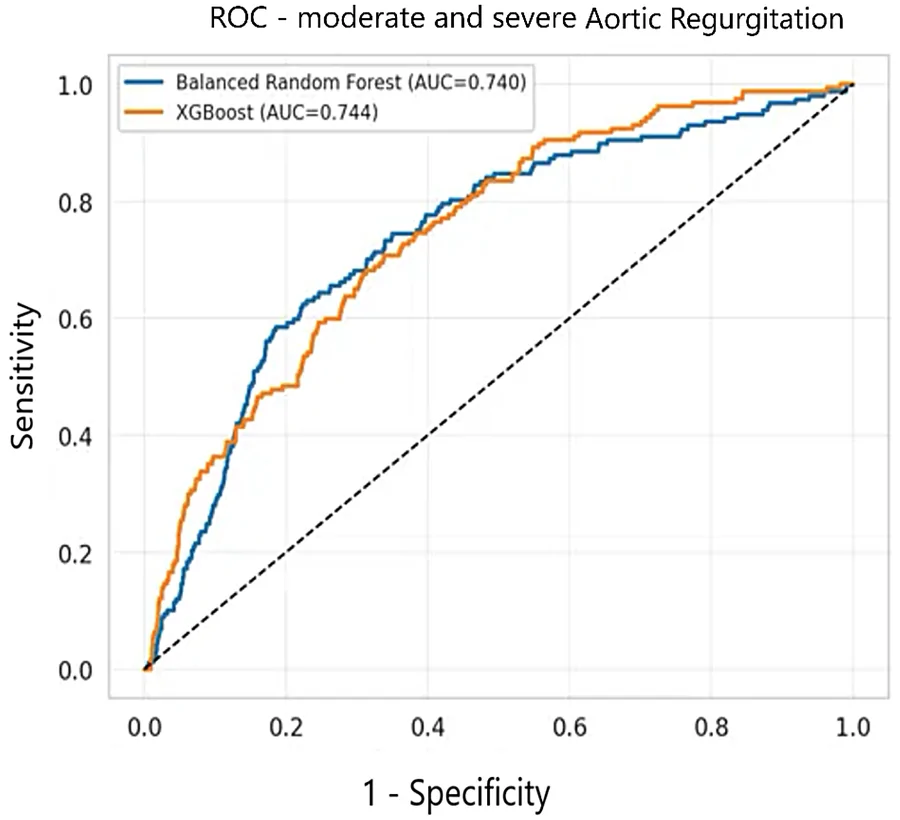
ROC curve. Moderate and severe aortic regurgitation.

**Figure 4:**
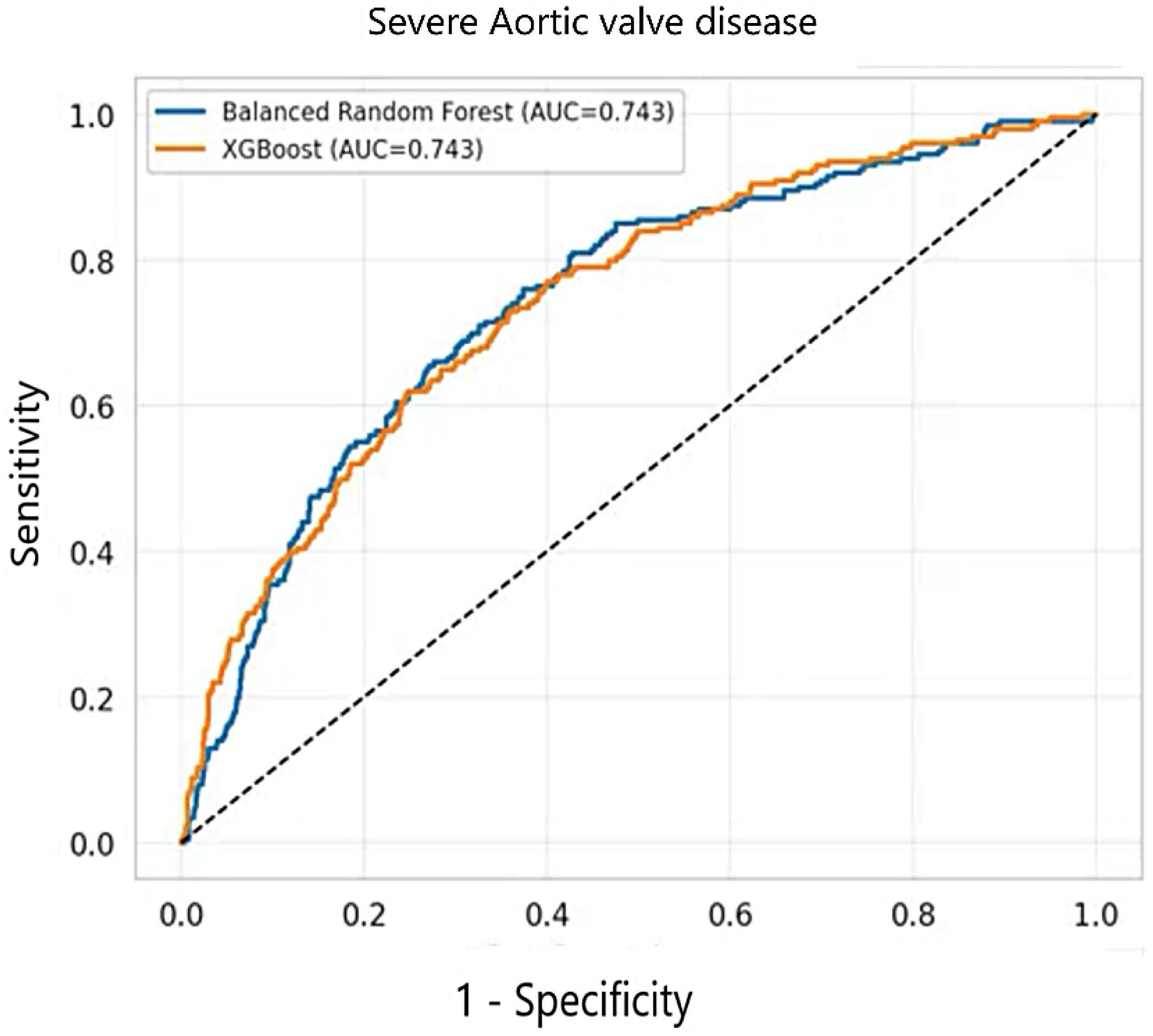
ROC curve. Severe aortic valve disease.

Sensitivity ranged from 0.579 to 0.611 and specificity from 0.650 to 0.734 across the three target variables at the Youden-optimal threshold, reflecting the trade-off between identifying patients and avoiding false positives. Fold-to-fold variability was substantial for moderate and severe aortic stenosis (Se 0.579 ± 0.342), the rarest target in the dataset (62/2503 positive cases, approximately six positive cases per validation fold), and comparatively modest for the composite target (Se 0.611 ± 0.135), which had a larger positive class Figures 4–7.

**Figure 5:**
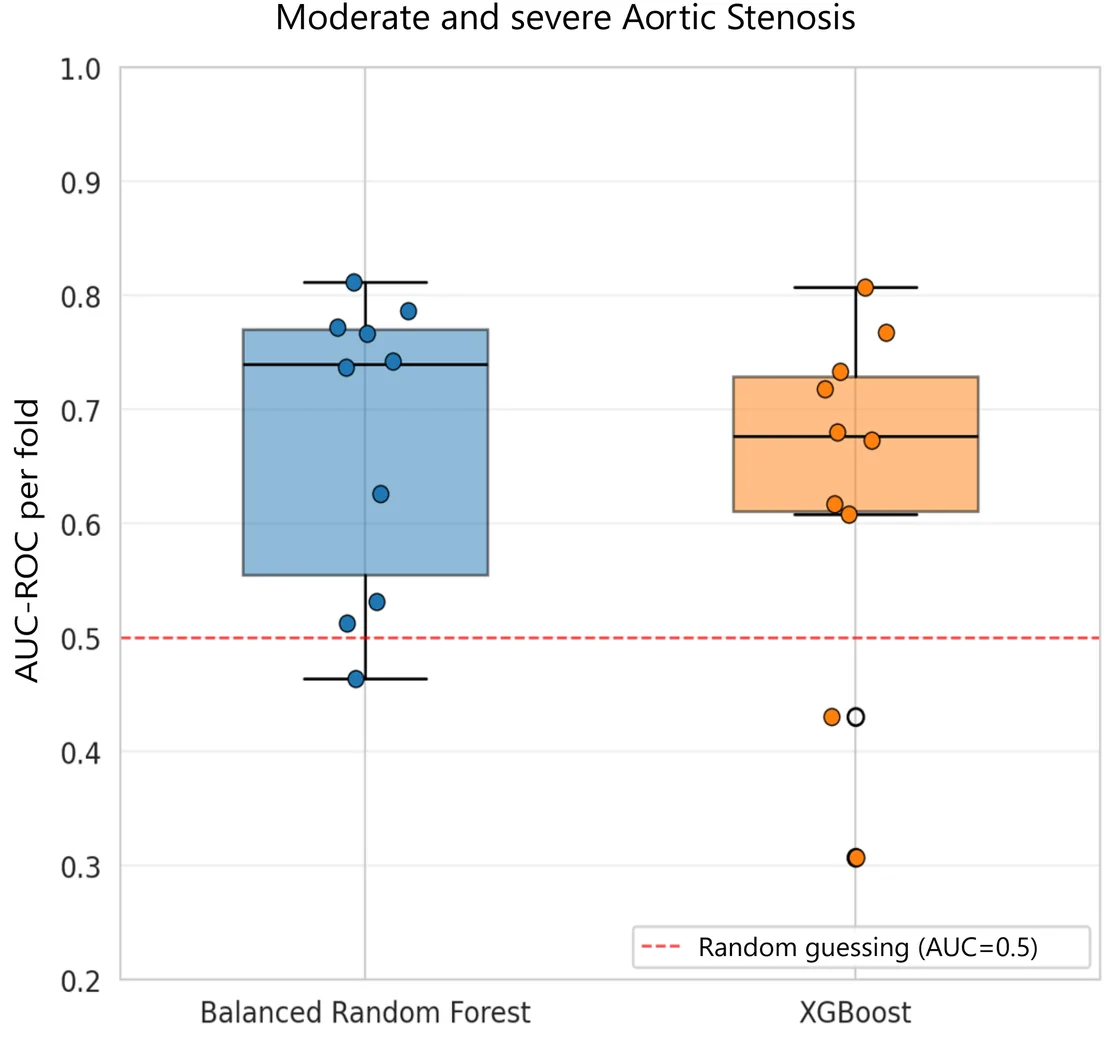
AUC stability per fold. Moderate and severe aortic stenosis.

**Figure 6:**
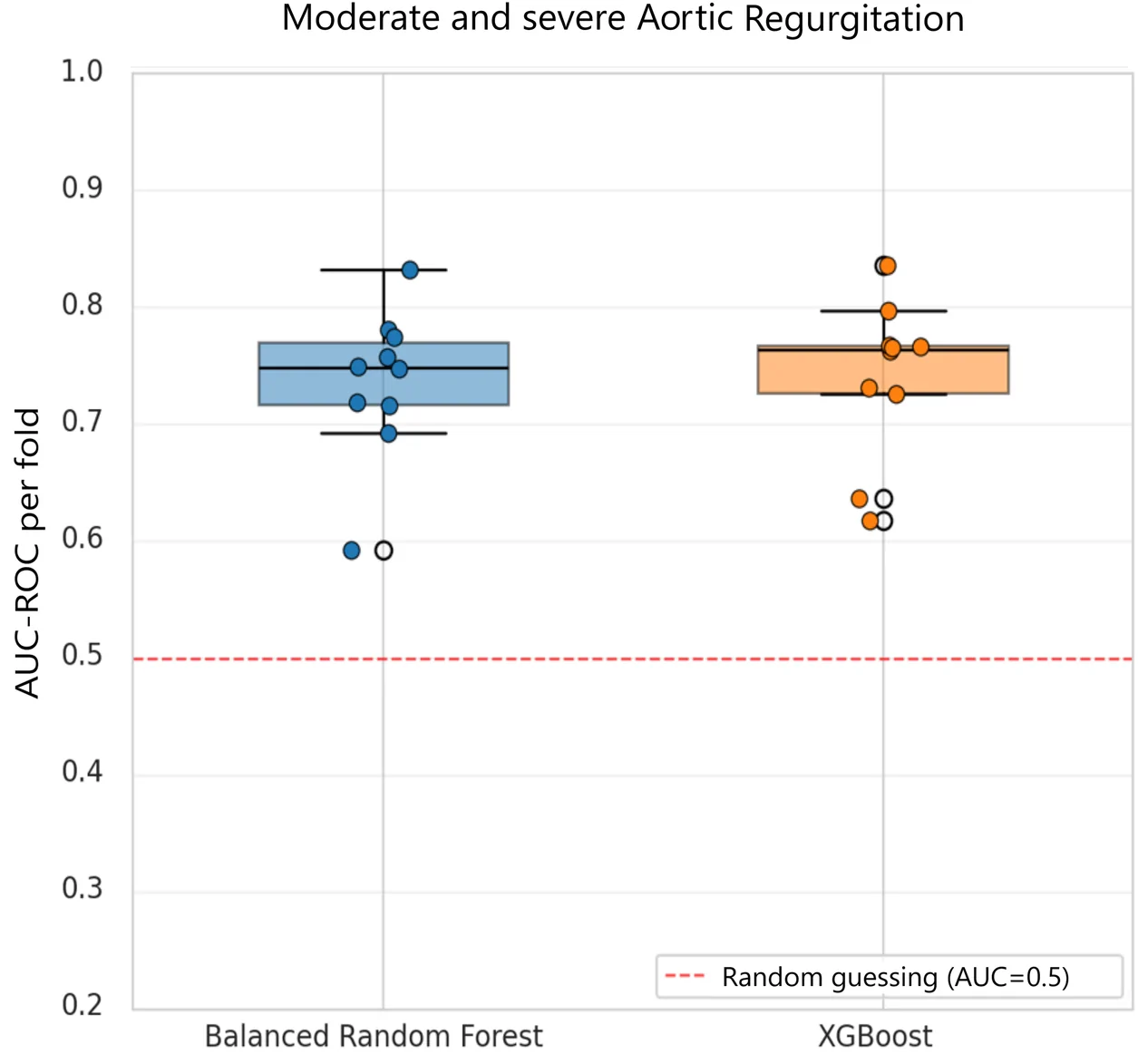
AUC stability per fold. Moderate and severe aortic regurgitation.

**Figure 7:**
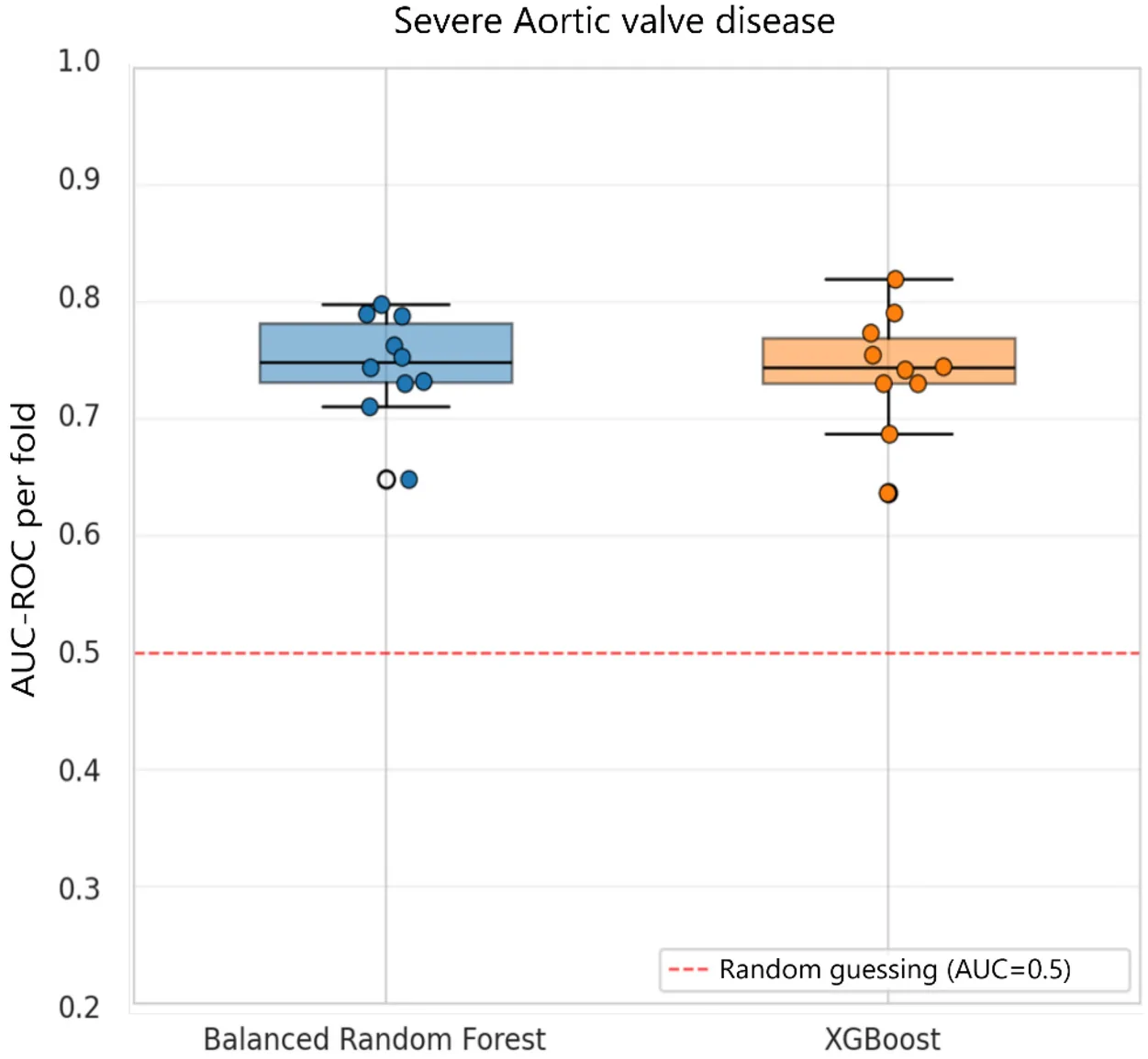
AUC stability per fold. Severe aortic valve disease.

The negative predictive value (NPV) was high across all targets (0.956–0.986), allowing for the confident exclusion of pathology even with a negative model result.

Optimal Youden thresholds were determined by nested cross-validation within the training portion of each fold and ranged from 0.505 to 0.517. A second operating point, fixing sensitivity at ≥90%, was also evaluated: at this threshold, sensitivity was 0.897–0.909 across targets, with specificity of 0.317–0.334 and NPV of 0.976–0.992, reflecting a configuration that minimizes missed cases at the cost of a higher referral rate for confirmatory echocardiography Table 3.

**Table 3:** Operating mode: sensitivity ≥90%.

Model	Sensitivity	Specificity	NPV
Balanced Random Forest	0.897 ± 0.079	0.334 ± 0.037	0.976 ± 0.017
XGBoost	0.885 ± 0.067	0.379 ± 0.048	0.977 ± 0.012

Results for significant Aortic valve disease (mean ± SD).

NPV, negative predictive value.

When recording the ECG, the patient's age, gender, and history of smoking and diabetes must be entered into the application. However, these parameters were not taken into account in this study; the analysis was based on single-channel ECG parameters.

### XGBoost model

AUC-ROC values ranged from 0.634 to 0.741, indicating a moderate ability of the model to discriminate between patients with and without pathology Table 2.

The highest values were obtained for the target “Aortic regurgitation” and the composite target “Significant aortic valve disease” (AUC-ROC = 0.741 for both), consistent with their larger positive class sizes Figures 2–4.

For the target “Aortic stenosis,” the model showed the lowest and least stable performance (AUC-ROC = 0.634 ± 0.155; Se = 0.464 ± 0.288, Sp = 0.665 ± 0.146), with two of ten validation folds falling below chance-level discrimination (AUC 0.307 and 0.430), reflecting the combined effect of the very low prevalence of this target (2.4%) and the sensitivity of gradient boosting to a small positive class. For the target “Aortic regurgitation,” a more balanced profile was achieved (Se = 0.617 ± 0.199, Sp = 0.719 ± 0.095). For the composite target, sensitivity was 0.678 ± 0.161 with a specificity of 0.692 ± 0.076. The negative predictive value (NPV) was high for all targets (0.962–0.982), allowing for the confident exclusion of pathology even with a negative model result.

At the alternative operating point fixing sensitivity at ≥90%, sensitivity ranged from 0.884 to 0.915 across targets, with specificity of 0.289–0.379 and NPV of 0.977–0.992 (Table 3).

The algorithm can be applied to the analysis of data from other single-channel ECG recorders after some modification, specifically regarding the ECG signal sampling rate.

## Discussion

Several recent publications have presented findings based on the analysis of electrocardiograms using artificial intelligence, confirming the considerable potential of this approach for screening and diagnosing prevalent, clinically significant cardiac abnormalities.

A report has been published on the use of an artificial intelligence-enabled 12-lead ECG algorithm for detecting undiagnosed severe AS, leading to transcatheter aortic valve replacement and altered perioperative management in an asymptomatic patient (15).

Another study (258,607 patients) found that 12-lead ECG analysis using artificial intelligence can identify patients with moderate or severe AS with an AUC of 0.85, sensitivity of 78%, specificity of 74%, and accuracy of 74% (16). In 2025, this model was tested in an Asian population. The AI-ECG model achieved an AUC of 0.85 (95% CI: 0.84–0.87) in detecting moderate to severe AS. Sensitivity was 0.83, specificity 0.65, PPV 0.37, NPV 0.94, and accuracy 68.29% (17).

Another international study demonstrated that AI-ECG models predicted future significant MR [0.774, 95% confidence interval (CI) 0.753–0.792], AR (0.691, 95% CI 0.657–0.720), and TR (0.793, 95% CI 0.777–0.808). For future significant MR, the hazard ratio (HR) was 7.6 (95% CI 5.8–9.9, *P* < 0.0001); for future AR, it was 3.8 (95% CI 2.7–5.5); and for TR, it was 9.9 (95% CI 7.5–13.0) (18).

All of the above articles analyzed 12-lead ECGs. Our study, however, used a single-lead ECG.

In our study, two machine learning models were developed and validated for predicting aortic valve pathology based on demographic and electrocardiographic parameters: Balanced Random Forest and gradient boosting (XGBoost). Both models were evaluated under an identical 10-fold group cross-validation protocol with leakage-free threshold selection, and both demonstrated moderate and closely comparable discriminatory ability.

AUC-ROC values ranged from 0.634 to 0.746 across the two models and three targets, indicating satisfactory ability to discriminate between patients with and without pathology; the difference between the two algorithms on the same targets did not exceed 0.04, within the range of fold-to-fold variability. The highest performance for both models was achieved for the composite target “Significant Aortic Valve Disease” (AUC-ROC = 0.746 for Balanced Random Forest, 0.741 for XGBoost), consistent with the larger size of this composite positive class. Balanced Random Forest showed more consistent performance across validation folds on the rarest target, moderate and severe aortic stenosis, whereas XGBoost showed wider fold-to-fold variability on this target, including two folds with below-chance discrimination. The high negative predictive value observed for both models (NPV 0.956–0.992 depending on target and threshold strategy) allows for the confident exclusion of significant disease in the presence of a negative result, which has considerable clinical significance for optimizing patient management.

A separate study assessed the impact of additional clinical comorbidity indicators (a history of myocardial infarction and any degree of anemia) on the Balanced Random Forest model. It was found that adding these parameters did not significantly impact quality indicators (AUC-ROC fluctuations within 0.01) or change the information content profile: none of these indicators were included among the final top 10 most significant predictors. This suggests that the hemodynamic consequences of a previous infarction and anemia are already indirectly reflected in high-frequency ECG parameters (QRSE, Sbeta, HFNoise) and rhythm variability indicators (SDNN, RR), which the model uses as its primary discriminant features.

The developed machine learning models demonstrate a moderate but statistically significant ability to predict significant aortic valve pathology based on parameters available in routine practice. Overall, the Balanced Random Forest model with these hyperparameters and 10-fold cross-validation demonstrated more consistent results and is recommended for use. The high negative predictive value (>96%) makes these models promising tools for pre-diagnostic exclusion and optimization of patient routing.

However, the model currently relies primarily on patient age, demonstrating a direct correlation between risk and aging. To create a more robust algorithm and improve the PPV in future studies, it would be desirable not only to increase the prevalence of the positive class in the training set but also to integrate other clinical and laboratory profiles that have independent prognostic value. It should be noted that the incidence of significant aortic valve disease in our sample was similar to that reported in various population-based studies. This allows us to use the obtained data for further research.

### Limitations

Our sample included relatively few patients with severe aortic valve disease. However, this study only demonstrates the feasibility and potential use of these methods for screening.

We did not test our models in an external group to assess their generalizability in this study. Additional testing is currently planned.

In this study, single-channel ECG devices from one manufacturer were used. Other single-channel ECG devices were not available for the current study.

Both models were evaluated using an identical 10-fold group cross-validation protocol, allowing direct comparison of their performance. However, the low prevalence of moderate and severe aortic stenosis in our cohort (2.4%) resulted in substantial fold-to-fold variability for both models on this target, and PPV remained low across all targets and both models (0.032–0.211) as an expected consequence of low disease prevalence rather than a model-specific limitation; external validation on an independent cohort would help establish the stability of these estimates.

## Conclusion

Single-lead ECG analysis using machine learning models demonstrated the potential of single-lead ECG in identifying patients with moderate to severe aortic valve disease with a diagnostic accuracy of 80%–82%.

## Data Availability

The datasets presented in this article are not readily available because the study used databases patented in the Russian Federation (patent numbers: No. 2021621563 and No. 2021621822). Requests to access the datasets should be directed to Natalia Kuznetsova, kuznetsova_n_o@staff.sechenov.ru.

## References

[1] Santangelo G Bursi F Faggiano A Moscardelli S Simeoli P Guazzi M. The global burden of valvular heart disease: from clinical epidemiology to management. J Clin Med. (2023) 12(6):2178. 10.3390/jcm12062178 36983180 PMC10054046

[2] Otto CM Nishimura RA Bonow RO Carabello BA Erwin JP Gentile F. 2020 ACC/AHA guideline for the man-agement of patients with valvular heart disease: a report of the American College of Cardiology/American Heart Association joint committee on clinical practice guidelines. J Thorac Cardiovasc Surg. (2021) 162(2):e183–353. 10.1016/j.jtcvs.2021.04.002 33972115

[3] Singh S Chaudhary R Bliden KP Tantry US Gurbel PA Visweswaran S. Meta-analysis of the performance of AI-driven ECG interpretation in the diagnosis of valvular heart diseases. Am J Cardiol. (2024) 213:126–31. 10.1016/j.amjcard.2023.12.015 38103769 PMC10842912

[4] Di Costanzo A Spaccarotella CAM Esposito G Indolfi C. An artificial intelligence analysis of electrocardiograms for the clinical diagnosis of cardiovascular diseases: a narrative review. J Clin Med. (2024) 13(4):1033. 10.3390/jcm13041033.1 38398346 PMC10889404

[5] Thaden JJ Nkomo VT Enriquez-Sarano M. The global burden of aortic stenosis. Prog Cardiovasc Dis. (2014) 56(6):565–71. 10.1016/j.pcad.2014.02.006 24838132

[6] Kuznetsova NO Alekseeva AM Mamedzade FE Sedov VP Kopylov FY Syrkin AL. Screening of heart defects in electrocardiogram analysis using machine learning methods (literature review) [in Russian]. J New Med Tech. e-edition. (2025) 19(1):44–54. 10.24412/2075-4094-2025-1-1-6

[7] Klimenko AA Andryashkina DY Karpova NY Kondrashov AA. Management of patients with aortic stenosis: what’s new in the 2023 clinical guidelines? RMJ. (2024) 3:2–6.

[8] Akinseye OA Pathak A Ibebuogu UN. Aortic valve regurgitation: a comprehensive review. Curr Probl Cardiol. (2018) 43(8):315–34. 10.1016/j.cpcardiol.2017.10.004 29174586

[9] Chiang S-J Daimon M Miyazaki S Kawata T Morimoto-Ichikawa R Maruyama M. When and how aortic stenosis is first diagnosed: a single-center observational study. J Cardiol. (2016) 68(4):324–8. 10.1016/j.jjcc.2015.10.007 26603322

[10] Kattoor AJ Shanbhag A Abraham A Vallurupalli S. Clinical context and detection of the murmur of advanced aortic stenosis. South Med J. (2018) 111(4):230–4. 10.14423/SMJ.0000000000000791 29719036

[11] Abe Y. Screening for aortic stenosis using physical examination and echocardiography. J Echocardiogr. (2021) 19(2):80–5. 10.1007/s12574-020-00511-8 33415574

[12] Davidsen AH Andersen S Halvorsen PA. Diagnostic accuracy of heart auscultation for detecting valve disease: a systematic review. Brit Med J Open. (2023) 13:e068121. 10.1136/bmjopen-2022-068121 PMC10040065 36963797

[13] Vahanian A Beyersdorf F Praz F Milojevic M Baldus S Bauersachs J. ESC/EACTS Scientific Document Group. ESC/EACTS guidelines for the management of valvular heart disease. Eur Heart J. (2022) 43(7):561–632. 10.1093/eurheartj/ehab395; Erratum in: Eur Heart J. 2022 Jun 1;43(21):2022. doi: 10.1093/eurheartj/ehac051 34453165

[14] Praz F Borger MA Lanz J Marin-Cuartas M Abreu A Adamo M. ESC/EACTS guidelines for the management of valvular heart disease. Eur Heart J. (2025) 46(44):4635–736. 10.1093/eurheartj/ehaf194; Erratum in: Eur Heart J. 2026 August 06:ehag625. doi: 10.1093/eurheartj/ehag625 40878295

[15] Tat E Oliver FR Malta P Castillo M Poterucha TJ Elias P. Artificial intelligence-enabled electrocardiographic detection of severe aortic stenosis leading to transcatheter aortic valve replacement. JACC Case Rep. (2026) 31(14):107184. 10.1016/j.jaccas.2026.107184 41778935 PMC13080615

[16] Cohen-Shelly M Attia ZI Friedman PA Ito S Essayagh BA Ko W-Y. Electrocardiogram screening for aortic valve stenosis using artificial intelligence. Eur Heart J. (2021) 42(30):2885–96. 10.1093/eurheartj/ehab153 33748852

[17] Kim D Lee E Kim J Kim EK Chang S-A Park S-J. External assessment of an artificial intelligence-enabled electrocardiogram for aortic stenosis detection. Eur Heart J Digit Health. (2025) 6(4):656–64. 10.1093/ehjdh/ztaf067 40703138 PMC12282354

[18] Liang Y Sau A Zeidaabadi B Barker J Patlatzoglou K Pastika L. Artificial intelligence-enhanced electrocardiography to predict regurgitant valvular heart diseases: an international study. Eur Heart J. (2025) 46(44):4823–37. 10.1093/eurheartj/ehaf448 40669514 PMC12634117

